# Demographic history of Ryukyu islanders at the southern part of the Japanese Archipelago inferred from whole-genome resequencing data

**DOI:** 10.1038/s10038-023-01180-y

**Published:** 2023-07-20

**Authors:** Kae Koganebuchi, Masatoshi Matsunami, Minako Imamura, Yosuke Kawai, Yuki Hitomi, Katsushi Tokunaga, Shiro Maeda, Hajime Ishida, Ryosuke Kimura

**Affiliations:** 1 Advanced Medical Research Center, Faculty of Medicine, University of the Ryukyus, Nishihara, 903-0215 Japan; 2 Department of Biological Sciences, Graduate School of Science, The University of Tokyo, Tokyo, 113-0033 Japan; 3 Department of Advanced Genomic and Laboratory Medicine, Graduate School of Medicine, University of the Ryukyus, Nishihara, 903-0215 Japan; 4 Division of Clinical Laboratory and Blood Transfusion, University of the Ryukyus Hospital, Nishihara, 903-0215 Japan; 5 Genome Medical Science Project, National Center for Global Health and Medicine, Tokyo, 162-8655 Japan; 6 Department of Microbiology, Hoshi University School of Pharmacy and Pharmaceutical Sciences, Tokyo, 142-8501 Japan; 7 Department of Human Biology and Anatomy, Graduate School of Medicine, University of the Ryukyus, Nishihara, 903-0215 Japan; 8 Mt. Olive Hospital, Naha, 903-0804 Japan

**Keywords:** Population genetics, Evolutionary biology

## Abstract

The Ryukyu Islands are located in the southernmost part of the Japanese Archipelago and consist of several island groups. Each island group has its own history and culture, which differ from those of mainland Japan. People of the Ryukyu Islands are genetically subdivided; however, their detailed demographic history remains unclear. We report the results of a whole-genome sequencing analysis of a total of 50 Ryukyu islanders, focusing on genetic differentiation between Miyako and Okinawa islanders. We confirmed that Miyako and Okinawa islanders cluster differently in principal component analysis and ADMIXTURE analysis and that there is a population structure among Miyako islanders. The present study supports the hypothesis that population differentiation is primarily caused by genetic drift rather than by differences in the rate of migration from surrounding regions, such as the Japanese main islands or Taiwan. In addition, the genetic cline observed among Miyako and Okinawa islanders can be explained by recurrent migration beyond the bounds of these islands. Our analysis also suggested that the presence of multiple subpopulations during the Neolithic Ryukyu Jomon period is not crucial to explain the modern Ryukyu populations. However, the assumption of multiple subpopulations during the time of admixture with mainland Japanese is necessary to explain the modern Ryukyu populations. Our findings add insights that could help clarify the complex history of populations in the Ryukyu Islands.

## Introduction

**Fig. 1:**
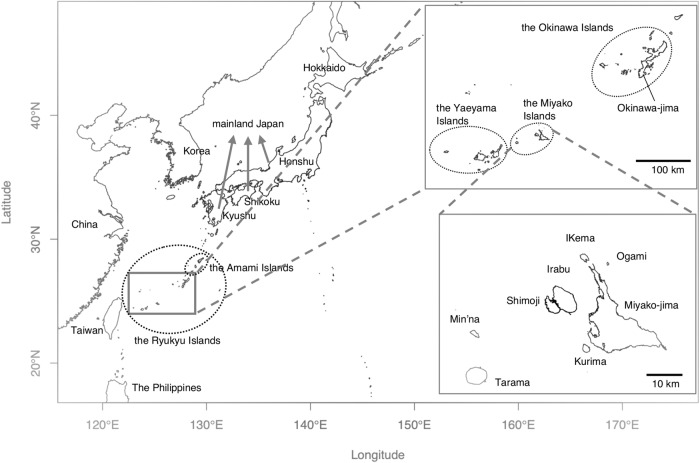
Map of the Japanese Archipelago and neighboring regions. The Japanese Archipelago consists of four main islands (Hokkaido, Honshu, Shikoku, and Kyushu), the Ryukyu Islands, and numerous other islands. The Ryukyu Islands consist of the Amami Islands, Okinawa Islands, Miyako Islands, and Yaeyama Islands. The Miyako Islands consist of Miyako-jima, Kurima, Ogami, Ikema, Irabu, Simoji, Min’na, and Tarama. The map was made by using Natural Earth (https://www.naturalearthdata.com) and the R maptools package

The Ryukyu Islands are a chain of islands distributed over 1000 km along the southernmost part of the Japanese Archipelago and divided into four primary island groups: the Amami Islands, the Okinawa Islands, the Miyako Islands, and the Yaeyama Islands (Fig. 1). The distances between Kyushu and the Okinawa islands, the Okinawa islands and the Miyako islands, the Miyako islands and the Ishigaki islands, and the Ishigaki islands and Taiwan are approximately 660, 290, 120, and 290 km, respectively. Archeological evidence has indicated that these island groups had different prehistoric cultures shaped by their own developments as well as outside influences.

In the Japanese Archipelago, Paleolithic skeletal remains have been recovered, mostly from fissures and caves in the Ryukyu Islands, such as the Minatogawa Fissure site (22,000–20,000 years before present [YBP]) [1, 2], the Sakitari Cave site (37,000–20,000 YBP) [3], the Pinza-abu Cave site (~29,000 YBP) [4], and the Shiraho-Saonetabaru Cave site (16,000 and 20,000 YBP) [5]. After the Paleolithic period, there was a period of time for which there is little evidence of habitation in the Ryukyu Islands until the beginning of the Shell-mound Period (6700–700 YBP) (Supplementary Fig. 1). Evidence of the Shell-mound Culture, which was affected by the Jomon Culture from the Kyushu region, has been found in the northern Ryukyus (the Amami Islands and the Okinawa Islands) but not the southern Ryukyus (the Miyako Islands and the Yaeyama Islands). Therefore, it has been suggested that a cultural boundary existed between the northern and southern Ryukyus during this period [6, 7]. In the southern Ryukyus, the Shimotabaru Culture (4200–3500 YBP) and the aceramic (non-pottery) culture (2500–900 YBP) have instead been identified. Between the two cultural periods, there was a temporal gap of approximately 1000 years. Although the source of these cultures in the southern Ryukyus is still debated, some archeologists have hypothesized that these cultures were influenced by the cultures of Taiwan and the northern Philippines [8, 9]. Around 800 YBP, the Gusuku Culture appeared in both the northern and southern Ryukyus. Rapid cultural changes were brought, probably by migrants from the main islands of Japan and by contacts with China [7]. The Gusuku Culture, which culturally unified the northern and southern Ryukyus, was characterized by the emergence of agriculture, the spread of ironware, and competitive polities. Subsequently, the Ryukyu Kingdom (600–200 YBP) was established and politically unified the Ryukyu Islands. The independent reign of the Ryukyu Kingdom ceased around 400 YBP because of domination by the Satsuma Fiefdom of southern Kyushu.

Numerous anthropological studies have attempted to reconstruct the history of the Japanese populations. Recent studies of ancient DNA have revealed that the Jomon people were split from a basal lineage of East Asians and isolated from the continental populations for a long period of time [10–12]. Accumulated evidence from morphological and genetic studies supports the hypothesis that the modern Japanese people were formed by a mixture of indigenous Jomon hunter-gatherers and migrants from continental East Asia during or after the Yayoi period [11, 13–19]. Genome-wide single-nucleotide polymorphism (SNP) and whole-genome sequencing data have shown that the modern Ryukyu islanders are genetically differentiated from the mainland Japanese (i.e., people of Honshu, Shikoku, and Kyushu Islands) [20–23]. It has been suggested that modern Ryukyu islanders received a greater genetic contribution from the Jomon people than did the mainland Japanese populations.

Previous genetic studies have also identified genetic differentiation among populations within the Ryukyu Islands [24–26]. In particular, genome-wide SNP data indicate that Miyako islanders can be genetically differentiated from Okinawa islanders and that genetic differentiation resulted primarily from the isolation between these populations rather than by gene flow from beyond the Ryukyu Islands, such as from the indigenous Taiwanese population [24]. The divergence time between Okinawa and Miyako islanders has been estimated at hundreds or at most thousands of years, suggesting that the Paleolithic people (~20,000 YBP) in the Ryukyu Islands are not the primary ancestors of modern Ryukyu islanders [24]. A more recent study examined population structures within the Miyako Islands, which were probably formed by multiple migrations from the Okinawa Islands [26]. However, the formation processes of the populations in Okinawa Islands and Miyako Islands have not sufficiently been validated in consideration of the admixture between Ryukyu Jomon people and migrants from mainland Japan after the Gusuku period.

In this study, we performed population genetic analyses using whole-genome resequencing (WGS) data to clarify the detailed demographic history of Ryukyu islanders. In the previous studies using SNP arrays, because of the ascertainment bias [27], it was difficult to accurately evaluate the nucleotide diversity and the site frequency spectrum, which are informative for population genetic analyses. WGS data are expected to provide a more accurate estimate of the demographic history of a population as they are free of ascertainment bias and contain information regarding rare variants. Our study, focusing on Okinawa and Miyako islanders, determined the processes of population formation and identified changes in the size of past populations. In particular, we examined how the two regions of the Ryukyu Islands differ in terms of genetic influences from the main islands of Japan.

## Materials and methods

### Subjects and genome sequencing

A total of 50 Ryukyu islanders (Okinawa, *n* = 25; Miyako, *n* = 25) participated in this study. Okinawans came from Okinawa Island. The Miyako islanders were identified from the Okinawa Bioinformation Bank and came from the four Miyako islands (Miyako-jima: *n* = 17; Ikema: *n* = 2; Irabu: *n* = 4; Tarama: *n* = 2). The geographic locations of each population sampled in this study are shown in Fig. 1. All subjects provided written informed consent to participate in this research. We confirmed by interview that all four grandparents of each participant were living on the respective islands. DNA samples were collected from blood or saliva of each participant. This study was approved by the ethical committees at the University of the Ryukyus and the University of Tokyo.

DNA was extracted from blood or saliva by SRL, Inc. (Tokyo, Japan). WGS was conducted at Macrogen Japan Corp. (Tokyo, Japan). DNA quality was assessed using the picogreen method, and the condition of the DNA was assessed by gel electrophoresis. A WGS library was constructed using a TruSeq DNA PCR-Free Library Preparation kit (Illumina Inc., San Diego, CA, USA) according to the manufacturer’s protocols. We sequenced the DNA using 2 × 150-bp paired-end reads on a HiSeq X sequencing platform (Illumina Inc.).

### Read processing and mapping

All paired-end reads were trimmed using Trimmomatic v.0.36 [28] with the following settings: ILLUMINACLIP: TruSeq3-PE.fa:2:30:10, LEADING:3, TRAILING:3, SLIDINGWINDOW:4:15, and MINLEN:36. Read files processing was conducted according to GATK Best Practices workflows for GATK 3.8. Trimmed reads were aligned to Genome Reference Consortium Human Build 37 (GRCh37), which was downloaded from the GATK resource bundle (https://software.broadinstitute.org/gatk/download/bundle) using BWA (v.0.7.16a-r1181) [29]. Duplicate reads were eliminated from BAM files using Picard tools (v.2.10.10) [30]. Base quality score recalibration was performed using GATK 3.8-1 to generate the final BAM files. Variants in each sample were called using GATK HaplotypeCaller, and per-sample gVCFs were then generated. Subsequently, joint genotyping was performed using GATK GenotypeGVCFs.

### Dataset processing and sample QC

We used variants with biallelic SNVs in which the proportion of missing data was <0.10. For WGS datasets, analyses were restricted to regions outside tandem repeats presented by the University of California Santa Cruz genome browser simpleRepeat table, and we excluded variant sites with a Hardy–Weinberg equilibrium (HWE) *p* < 10^−2^ in all populations. The *p* value for filtering was determined based on the inflation of HWE *p* values using quantile-quantile plots. Finally, we found 5,863,618 variants from whole-genome sequencing and joint variant calling in Ryukyu Islanders, Honshu, and Han.

We calculated the inbreeding coefficients (*F*) and the pairwise IBD estimation (pi_hat) using plink 1.9 [31]. There were no individuals with *F* > 0.0625 or pi_hat >0.125. Therefore, all samples were used for the analysis.

### Genetic statistics and annotation

In estimating genetic statistics, we used only SNVs that were variable among Han Chinese and had a minor allele frequency >0.05 in each population in order to avoid overestimating genetic differences between populations. The pairwise weighted mean *F*_ST_ was calculated using the snpStats package [32].

### Population structure analysis

PCA was performed using the smartpca program in EIGENSOFT (v.7.2.1) [33]. Population clustering was confirmed via k-means clustering on PC1 and PC2. We used East and Southeast Asian genome sequencing data from the Simons Genome Diversity Project [34] as a reference panel. Additionally, to identify the extent of genetic ancestry in each individual, model-based clustering was conducted using ADMIXTURE [35]. We tested different numbers of clusters, from *K* = 2 to *K* = 4.

### Demographic inferences

*TreeMix* [36] was used to infer a bifurcating population tree and admixture events. The VCF file was converted to *TreeMix* format using plink1.9 [37] and a python script, plink2treemix.py [38]. We fitted 0–2 admixture edges using the five clusters estimated from PCA and cluster analysis. Han Chinese was set as an outgroup. ƒ_3_ and ƒ_4_ statistics were computed using the AdmixTools package [39] to estimate affinities among populations using the program *qp3Pop* with default parameters and *qpDstat* with f4mode: Yes. Standard errors were computed using a block jack-knife (significance cut-off of |*Z*| > 3).

Admixture graph modeling was carried out using *qpGraph* (useallsnps: YES, blgsize: 0.05, forcezmode: YES, diag: 0.0001, bigiter: 6, hires: YES, lambdascale: 1, inbreed: YES) from the AdmixTools package [39]. The program is used to fit admixture graphs by computing the allele-sharing summary statistics known as *f*-statistics [40]. An admixture graph consists of a sequence of population splits, admixture event locations, branch length parameters in units of genetic drift, and admixture proportions. The program returns a list of residual poorly predicted f-statistics and their *z*-scores, which provide information about the performance of the model. In the *qpGraph* analysis, we used the genome data of Hokkaido Jomon (F23) [11] in addition to Han Chinese, Honshu, OK, S-MY and N-MY. Because we included the ancient DNA data, the analysis was performed using all the SNVs or only transversions (1,885,361 SNVs). We modeled population histories including previously documented possible admixture events, assuming ancient Ryukyu populations: Ryukyu_Jomon, Okinawa_Jomon, Miyako_Jomon, Okinawa_Gusuku, and Miyako_Gusuku. We added all possible edges in the graph and retained only the estimated graph that provided no individual ƒ-statistics with |*Z*| > 3 between empirical and predicted statistics.

The effective size of each population was estimated using WGS data and SMC++ [41]. The VCF file was converted to SMC format using the vcf2smc option in the SMC++ program for all individuals of each cluster separately. As recommended by the SMC++ tutorial, unmapped regions of the genome were masked using the vcf2smc command with the –m flag. We fixed the mutation rate at 1.25 × 10^−8^ per generation per base pair. In order to verify the effect of differences in the number of samples, datasets in which five samples were randomly extracted 10 times in Han Chinese, Honshu, OK, and S-MY populations were prepared, and the same estimation was performed.

## Results

### Genetic variation in WGS data

We obtained in total 50 WGS datasets from Okinawa (*n* = 25) and Miyako (*n* = 25) islanders at an average depth of 36.3× (max: 46.9×, min: 29.4×). We also used the data for Honshu Japanese (*n* = 25) [42, 43] and Han Chinese (*n* = 25) obtained from deep sequencing data of the 1000 Genomes Project Chinese Han in Beijing (CHB) [44]. We called variants jointly using GATK3 for population genetic analyses and identified 5,863,618 single-nucleotide variants (SNVs).

We calculated the weighted mean *F*_ST_ for all pairs of the three Japanese populations (Okinawa, Miyako, and Honshu). Honshu vs. Miyako exhibited a higher *F*_ST_ value (4.70 × 10^−3^) than Honshu vs. Okinawa (2.79 × 10^−3^), and Okinawa vs. Miyako (2.25 × 10^−3^) exhibited the lowest *F*_ST_ value, indicating that the pattern was consistent with isolation by distance (Supplementary Fig. 2).

### Population structure of Ryukyu islanders

**Fig. 2:**
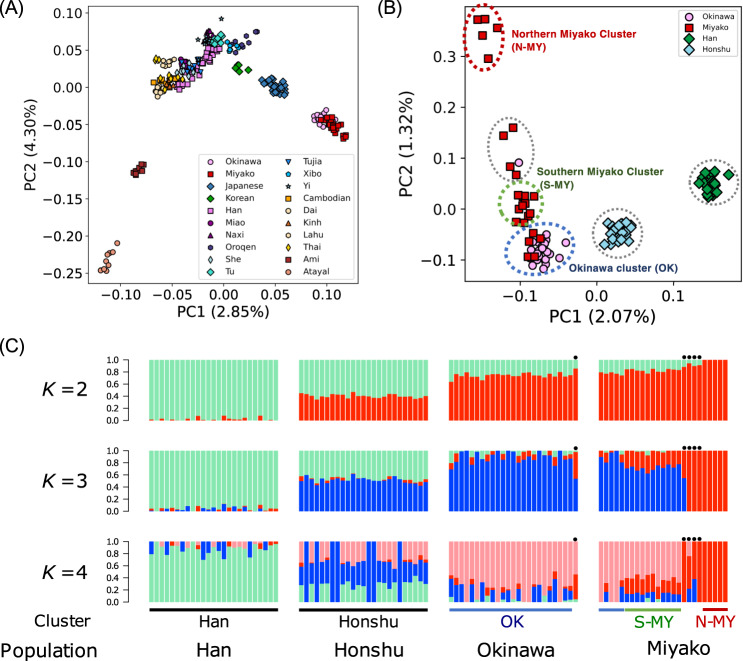
Results of PCA and ADMIXTURE analysis using WGS data. **A** PC1 vs. PC2 based on a PCA with reference microarray dataset of East and Southeast Asian populations from the Simons Genome Diversity Project. **B** PC1 vs. PC2 based on a PCA including Han Chinese, Honshu, Okinawa, and Miyako individuals. Dashed circles represent clusters based on the *k*-means method (*k* = 6). Clusters for northern Miyako (N-MY; red), southern Miyako (S-MY; green), and Okinawa (OK; blue) were formed. **C** Stacked barplots showing individual ancestries estimated by ADMIXTURE. “Cluster” denotes a cluster based on PCA (**B**). Black dots indicate individuals placed between N-MY and S-MY in (**B**). Cross-validation errors were 0.518 for *K* = 2, 0.539 for *K* = 3, and 0.568 for *K* = 4

We examined the genetic structure patterns in East and Southeast Asian populations by principal component analysis (PCA), jointly analyzing a publicly available microarray dataset from the Simons Genome Diversity Project (Fig. 2A). PC1 distinguished Ryukyu (Okinawa and Miyako) islanders from the other East and Southeast Asian populations. The population closest to Ryukyu islanders in the PC1 axis was the mainland Japanese, followed by the Korean and Chinese populations. However, Taiwan aborigines were genetically distant from the Ryukyu islanders, even though these populations are geographically very close. The pattern of the PC1-PC2 plot exhibited an inverse “U” shape, a pattern usually observed in stepping-stone models of populations [45]. Therefore, the distance in PC2 may not provide any direct information regarding the genetic distance between populations.

We then conducted PCA of Okinawa and Miyako islanders, Honshu Japanese, and Han Chinese (Fig. 2B). After k-means clustering (*k* = 6), both the Han Chinese and Honshu Japanese formed an independent cluster. We identified four clusters for the Ryukyu populations. Twenty-four Okinawa islanders formed a cluster together with five Miyako islanders, which we designated the Okinawa (OK) cluster (blue circle in Fig. 2B), suggesting that these five Miyako individuals were recent migrants from the Okinawa Islands. The cluster next to the OK cluster was composed of 11 Miyako islanders. According to the questionnaire, these individuals were from Miyako-jima and Tarama in the southern part of the Miyako Islands (the southern Miyako [S-MY] cluster; green circle in Fig. 2B). The five Miyako islanders, who were from Ikema and Irabu in the northern region of the Miyako Islands, clustered at an extremely distant position relative to the others (the northern Miyako [N-MY] cluster; red circle in Fig. 2B). One Okinawa islander and four Miyako islanders were plotted between the N-MY and S-MY clusters, suggesting that these individuals were mixed between the two different regions in the Miyako Islands. This observation was consistent with the results of the questionnaire survey in terms of the birthplaces of these individuals’ grandparents, except for the one Okinawa islander.

In the ADMIXTURE analysis of all Ryukyu islanders, the *K* value providing the lowest cross-validation error was *K* = 2, in which the Han Chinese formed one extreme (green), N-MY formed another extreme (red), and the remaining individuals were intermediate (Fig. 2C). When we assumed *K* = 3, an ancestral component for the Okinawa islanders (blue) appeared, and individuals in the S-MY cluster seemed to form a mixture between the two components.

### Population history of Ryukyu islanders

**Fig. 3:**
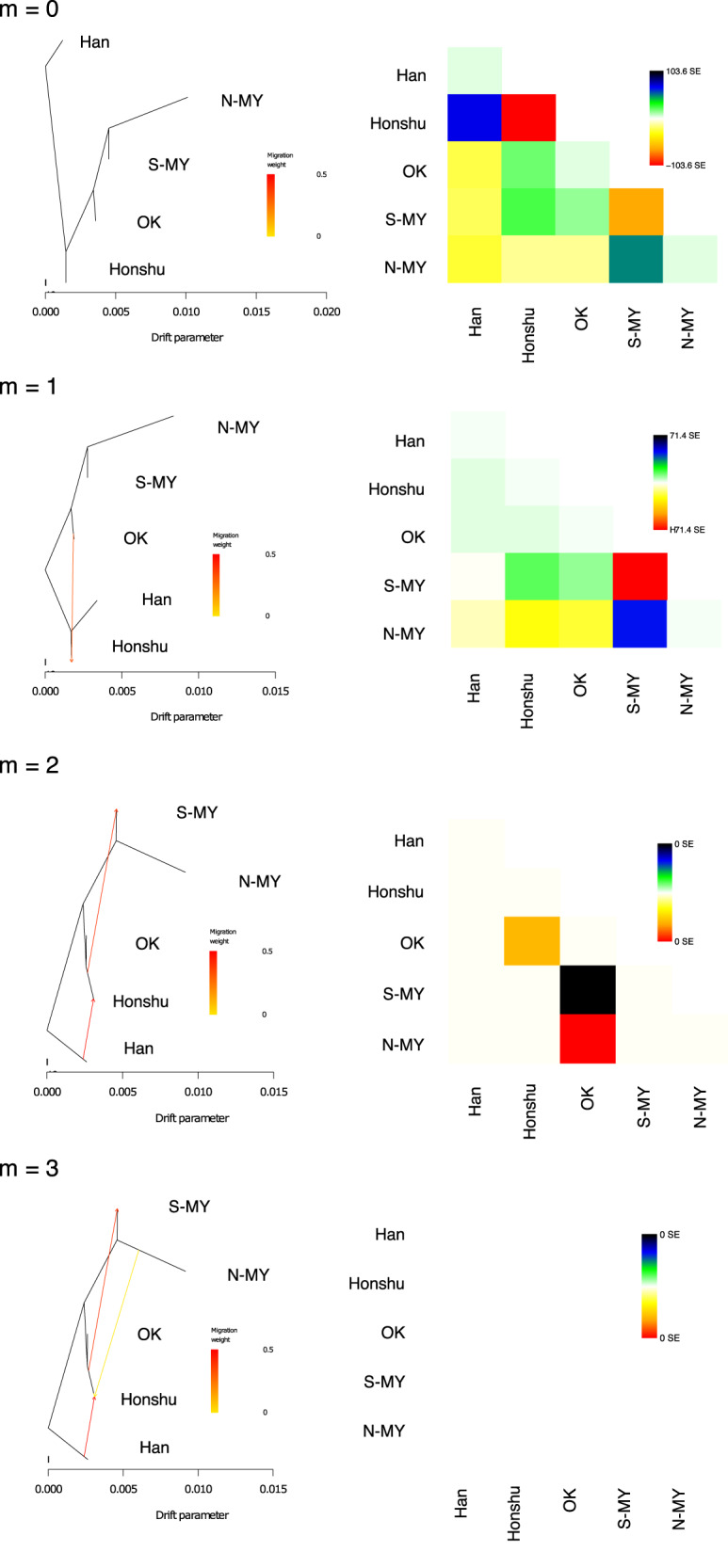
*TreeMix* results. Phylogenetic trees and matrix of residuals among the five clusters based on a model without admixture (*m* = 0) and models with 1, 2, and 3 migration edges (*m* = 1–3). Arrows indicate migration edges

To estimate the phylogenetic relationship and past migration events among the populations, we performed a *TreeMix* analysis, regarding the clusters as populations. Four graphs with 0–3 migration edges are shown in Fig. 3. We found that the N-MY cluster had a relatively long branch length, indicating that a strong genetic drift occurred in this population. The high values of residuals suggested that a tree model without migration (*m* = 0) and a model with one migration (*m* = 1) would fit the data poorly. A model with two migrations (*m* = 2) or more (*m* = 3) provided a better fit to the data. In *m* = 2 and 3, the migration edge from Han Chinese to Honshu Japanese likely indicated migration from continental East Asia to Japan, primarily during the Yayoi period. However, the other migration edges could not be simply interpreted according to our historical and archeological knowledge: a migration edge started at the split point of Honshu and the OK cluster and ended at the S-MY cluster (*m* = 2 and 3), whereas another edge started at the branch for the N-MY cluster and ended at the Honshu Japanese (*m* = 3). The real demographic history may be more complex and the *TreeMix* analysis can underestimate the number of migration edges and simplify the model [36]. In such a case, the topology and lengths of branches can be distorted and unrealistic migration edges can be drawn to minimize the residuals. Therefore, we need to carefully interpret the results of *TreeMix*.

**Fig. 4:**
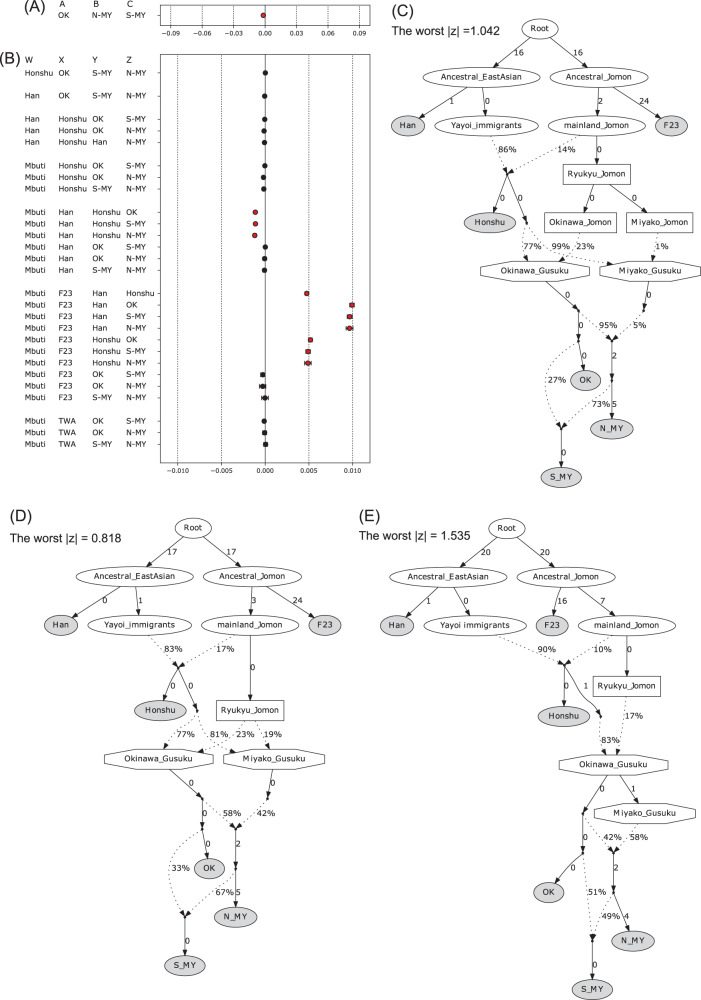
Modeling of the admixture among the populations. **A** ƒ_3_ statistics. A negative value of ƒ_3_ (X; Y, Z) provides proof that population X was admixed between populations Y and Z. **B** ƒ_4_ statistics. A positive (negative) value of ƒ_4_ (W, X; Y, Z) indicates gene flow from population X into population Z (Y). Whiskers represent standard error. Statistics with |*Z*| > 3 are highlighted in red. **C**–**E** Admixture graph modeling for Ryukyu islanders. We assumed three demographic models: **C** two panmictic populations, Okinawa_Jomon and Miyako_Jomon, mixed with people from the main islands of Japan. **D** A panmictic Ryukyu_Jomon population mixed with people from the main islands of Japan and Okinawa and Miyako independently. **E** A panmictic Ryukyu_Jomon population mixed with people from the main islands of Japan and Okinawa, and Miyako_Gusuku split from the admixed population. Solid arrows indicate drift edges shown in units of *F*_ST_ × 1000. Dashed arrows indicate admixture edges and mixture proportions. Gray circles denote actual populations (or individuals) as input data, whereas white circles denote hypothetical populations. Box nodes indicate populations of the Ryukyu Islands that originated from Jomon people of the main islands of Japan. Octagon nodes indicate populations derived from admixture between the Ryukyu Jomon population and people from the main islands of Japan

To further assess the gene flow among the populations, we tested the data using ƒ_3_ and ƒ_4_ statistics. The test of ƒ_3_(S-MY; N-MY, OK) returned a significantly negative value (Fig. 4A), suggesting that the S-MY population was formed by an admixture between the N-MY and OK populations. However, ƒ_4_(Honshu, OK; S-MY, N-MY) and ƒ_4_(Han, OK; S-MY, N-MY) tests did not significantly deviate from zero (Fig. 4B), indicating that the interaction between the OK and S-MY populations was not significant in these tests. When we tested ƒ_4_(Han, Honshu; Y, Z) ƒ_4_(Mbuti, Honshu; Y, Z), and ƒ_4_(Mbuti, Han; Y, Z), where Y and Z represent the Ryukyu populations, the statistics did not return any significant values. The value of ƒ_4_(Mbuti, Han; Honshu, Z), where Z represents a Ryukyu population, was significantly negative, indicating that Honshu had a greater genetic influence from the continental East Asian population than did the Ryukyu populations.

The genetic contribution from Jomon people was also tested using ƒ_4_ statistics. When we included the Jomon data (F23) [11] (i.e., ƒ_4_[Mbuti, F23; Y, Z]), a significant value of the statistic would signal a difference between the Y and Z populations in terms of the genetic contribution rate of the Jomon people. The results demonstrated that the genetic influence of the Jomon people upon the Ryukyu populations was larger than that upon Honshu Japanese (Fig. 4B). In addition, the results also indicated no significant difference in the genetic influence of the Jomon people upon the three Ryukyu populations. These results suggest that the genetic differentiation between the Okinawa and Miyako populations formed independently of the genetic differentiation between the Jomon people and Yayoi migrants or between the Honshu Japanese and Ryukyu populations.

We also tested the possibility that gene flow from the Taiwanese aborigines had caused the genetic differentiation between the Okinawa and Miyako populations. The value of ƒ_4_ (Mbuti, TWA; Honshu, Z), where Z represents a Ryukyu population, did not significantly deviate from zero (Fig. 4B). This result suggests that gene flow from the Taiwanese aborigines is not the primary reason for the genetic differentiation among Ryukyu populations.

To explore how the Ryukyu populations formed, we modeled past population splits and mixtures using *qpGraph*. Based on archeological and anthropological evidence, we hypothesized more complex models than those estimated by the TreeMix analysis, in which the modern Ryukyu populations formed via admixture between indigenous hunter-gatherers in the Ryukyu islands (Ryukyu Jomon) and migrants from the main islands of Japan before or during the Gusuku period and through subsequent interactions between the populations. Three different scenarios were considered in this analysis. The first scenario assumed that Ryukyu Jomon populations differentiated between the ancestors of Miyako and Okinawa islanders (Fig. 4C). In the second scenario, there was only one Ryukyu Jomon population, and the admixtures between the Ryukyu Jomon and mainland Japanese migrants in the early phase of the Gusuku period differed between Okinawa islanders and Miyako islanders (Fig. 4D). The third scenario hypothesized that there was only one panmictic population at the time of admixture with mainland Japanese, and then the populations diverged (Fig. 4E). As results, all of the models were acceptable, showing the worst |*z*| score of less than 3, and the second model (Fig. 4D) had the smallest value of the worst |*z*| score (0.818). Additionally, in the first model (Fig. 4C), both the drift parameters from Ryukyu Jomon to Okinawa Jomon and to Miyako Jomon were 0, which indicated that this model is substantially the same as the second model. Therefore, we considered that the second model best fit the data. Although the third model (Fig. 4E) was not rejected, our results suggested that the assumption of one Ryukyu Jomon population is sufficient. The estimation using both transversions and transitions also suggested the second was the best model (Supplementary Fig. 3B: the worst |*Z*| = 1.263).

**Fig. 5:**
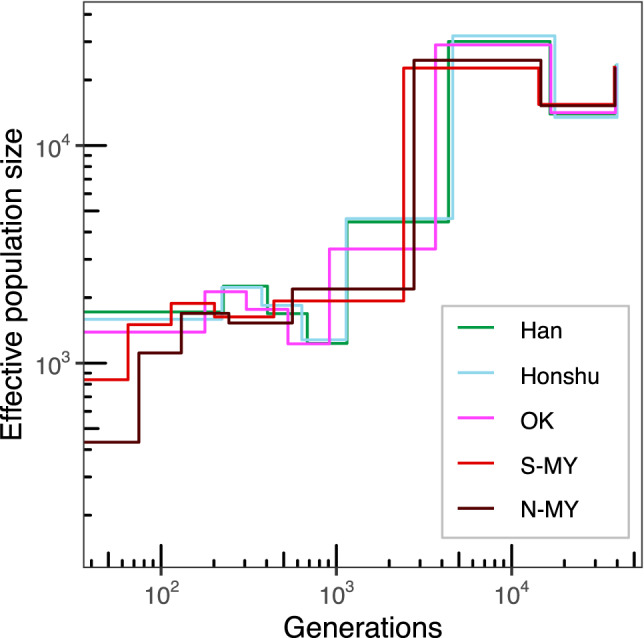
Dynamics of effective population sizes of the five clusters inferred using SMC++

Finally, to elucidate changes in effective population size, we applied the SMC++ method, which relies on the site frequency spectrum. Based on the trajectories of past effective population size (Fig. 5), all five populations were inferred to have experienced an out-of-Africa bottleneck approximately 2000–5000 generations ago. After the event, the Han, Honshu, and OK clusters almost maintained their population size. In contrast, the population sizes of the N-MY and S-MY clusters declined approximately 70 generations ago. The degree of reduction was greater in the N-MY cluster than S-MY cluster. In an analysis using down-sampled datasets, five individuals each of the Han, Honshu, OK, and S-MY clusters did not show any tendency toward recent population size reduction, except for S-MY (Supplementary Fig. 3). Therefore, these data suggest that the small number of samples in the N-MY cluster is not the reason for the population size reduction in the analysis.

## Discussion

Elucidating the population structure and history of Ryukyu islanders is of great interdisciplinary interest. The present study examining WGS data confirmed a genetic differentiation between Okinawa islanders and Miyako islanders [24, 25, 46]. The PCA and ADMIXTURE analysis patterns (Fig. 2) revealed a genetic cline in the Ryukyu Islands, which was observed in a previous study using SNP microarray data [26].

The population differentiation in the Ryukyu Islands could have been primarily caused by genetic drift rather than a difference in gene flow from beyond the Ryukyu Islands, such as from the main islands of Japan and Taiwan [24]. Our study also supports this possibility. A reduction in the population size of the N-MY cluster was inferred from the long branch length in *TreeMix* (Fig. 3), the values of the drift edges that went to N-MY in *qpGraph* (Fig. 4C–E), and by past demographic fluctuation in SMC++ (Fig. 5). This population size reduction was consistent with the previous estimation using SNP array data based on identical-by-descent segments [26], although our SMC++ analysis did not detect population increase in recent times. Furthermore, we did not find any significant evidence indicating a difference in the degree of gene flow from the mainland Japanese or from Taiwanese aborigines (Fig. 4B). In the Miyako Islands, an aceramic culture was identified before the Gusuku period (Supplementary Fig. 1). The origin of the people who developed the aceramic culture is still unknown, although some archeologists have postulated that this culture shares some common points with the Austronesian cultures of Taiwan and the northern Philippines [9]. A recent study of ancient DNA suggested that prehistoric individuals from the Nagabaka site during the aceramic period in Miyako-jima had Jomon genetic ancestry [47]. Our study did not identify the people who developed the aceramic culture, but our data do not support the idea that Taiwanese aborigines were involved in the formation of the modern Miyako islanders.

The results of ƒ_4_ statistic analyses revealed a slight non-significant difference between the Ryukyu populations in terms of the genetic contribution from Jomon hunter-gatherers (Fig. 4B). The admixture graph (Fig. 4D) also suggested that the admixture rate with people from the main islands of Japan differed slightly between the northern and southern Ryukyu islanders (77% and 81%, respectively). This result was unexpected because the Miyako Islands are geographically farther from the main islands of Japan than are the Okinawa Islands.

The admixture graph (Fig. 4D) further demonstrated that mainland Japanese were formed via admixture between mainland_Jomon (17%) and Yayoi immigrants (83%). Thus, considering admixture between the mainland Japanese and Ryukyu_Jomon, we calculated the total Jomon genetic component among the Ryukyu islanders as ~36%, a value 1.4 times that of the mainland Japanese (17%). Previous studies have estimated the proportion of Jomon ancestry among mainland Japanese and Ryukyu islanders [12, 14, 16, 18, 48]. Kanzawa-Kiriyama et al. [11] reported that the genetic contributions of the Jomon people were 9% (*qpGraph*) and 13.7% (ƒ_4_ ratio test) among modern Japanese and 27.4% (ƒ_4_ ratio test) among Ryukyu islanders, considering the CHB as another source population. Our estimated admixture rates were higher than those in the previous study; the difference in the estimated rate may be dependent on referred populations and assumed models.

There are various possible scenarios for peopling of the Ryukyu Islands. One possibility is that the people who developed the aceramic culture in the Miyako Islands originated from the Ryukyu Jomon as a recent ancient DNA study suggested [47] and genetically contributed to the modern Miyako islanders. Another point to be considered is whether there were multiple subpopulations in the early phase of the Gusuku period. To consider these points, we examined three scenarios for the formation of the Ryukyu populations using the admixture graph. Our analysis suggested that there were multiple subpopulations during the early phase of the Gusuku period (Fig. 4D) and rejected the scenario that assumes a panmictic population at that time (Fig. 4E). Our analysis also indicated that an assumption of multiple subpopulations of Ryukyu Jomon (Fig. 4C) is not necessary. This means that the people who developed the aceramic culture, in either case of their origin, would not have made a large genetic contribution to the modern Miyako islanders. Finally, we accept the model shown in Fig. 4D, which indicates that the admixture of a panmictic Ryukyu Jomon population with migrants from the main islands of Japan occurred in different ways between the ancestors of the Okinawa and the Miyako populations. It should be noted that it is not necessary that the admixture of ancestors of the Miyako population occurred in the Miyako Islands. Considering archeological and historical evidence, it can be hypothesized that there were several subpopulations in the Okinawa Islands and that the Miyako islanders were derived from one of these subpopulations.

It is also notable that genetic differentiation was observed between the N-MY and the S-MY clusters, which are geographically close. Individuals in the N-MY cluster reside on Ikema and Irabu, whereas individuals in the S-MY cluster reside on Miyako-jima and Tarama. Historically, the people of Ikema have been isolated from people in other areas, and the people of Irabu derived from the people of Ikema [49, 50]. Evidence for limited interactions between the areas within the Miyako Islands can also be seen in the diversity of dialects [51]. There are several dialects in the Miyako Islands, and the Ikema dialect spoken on Ikema Island differs substantially from the other Miyako dialects. These genetic and linguistic differentiations may have been caused by geographical, cultural, and/or political isolation.

Matsunami et al. [26] suggested that there were multiple migrations from the Okinawa Islands to the Miyako Islands. In our models used to generate the admixture graph (Fig. 4C–E), two relatively recent migrations from the Okinawa Islands to the Miyako Islands were assumed. In the accepted model for the formation of the S-MY population (Fig. 4D), the admixture rates from the Okinawa Islands were 58% in the first migration and 33% in the second migration. The genetic cline observed among Ryukyu islanders can be explained by such recurrent migrations beyond regional boundaries.

In conclusion, we identified a genetic cline among Ryukyu Islanders that was shaped primarily by genetic drift that occurred in a subpopulation of the Miyako Islands and by interregional migrations. However, questions remain regarding the details of the population history of the Ryukyu Islands. For example, where did the Paleolithic people of the Ryukyu Islands originate? How were the Shimotabaru and aceramic cultures formed? Comprehensive studies including analyses of ancient and modern genomes would be helpful to clarify the detailed population history of the Ryukyu Islands and add new insights regarding human activities on the islands of East Asia.

### Supplementary information

Supplementary figures 1-4

## Data Availability

The data underlying this article cannot be shared publicly for the privacy of individuals that participated in the study. The data will be shared on reasonable request to the corresponding author.
