# Supplementary material for: Demographic history of Ryukyu islanders at the southern part of the Japanese Archipelago inferred from whole-genome resequencing data
Source: J Hum Genet. 2023 Jul 20;68(11):759–67. doi: 10.1038/s10038-023-01180-y (PMC10597838; doi:10.1038/s10038-023-01180-y)
Supplement: Supplementary file 1 — Supplementary figures 1-4 [file 10038_2023_1180_MOESM1_ESM.pdf]

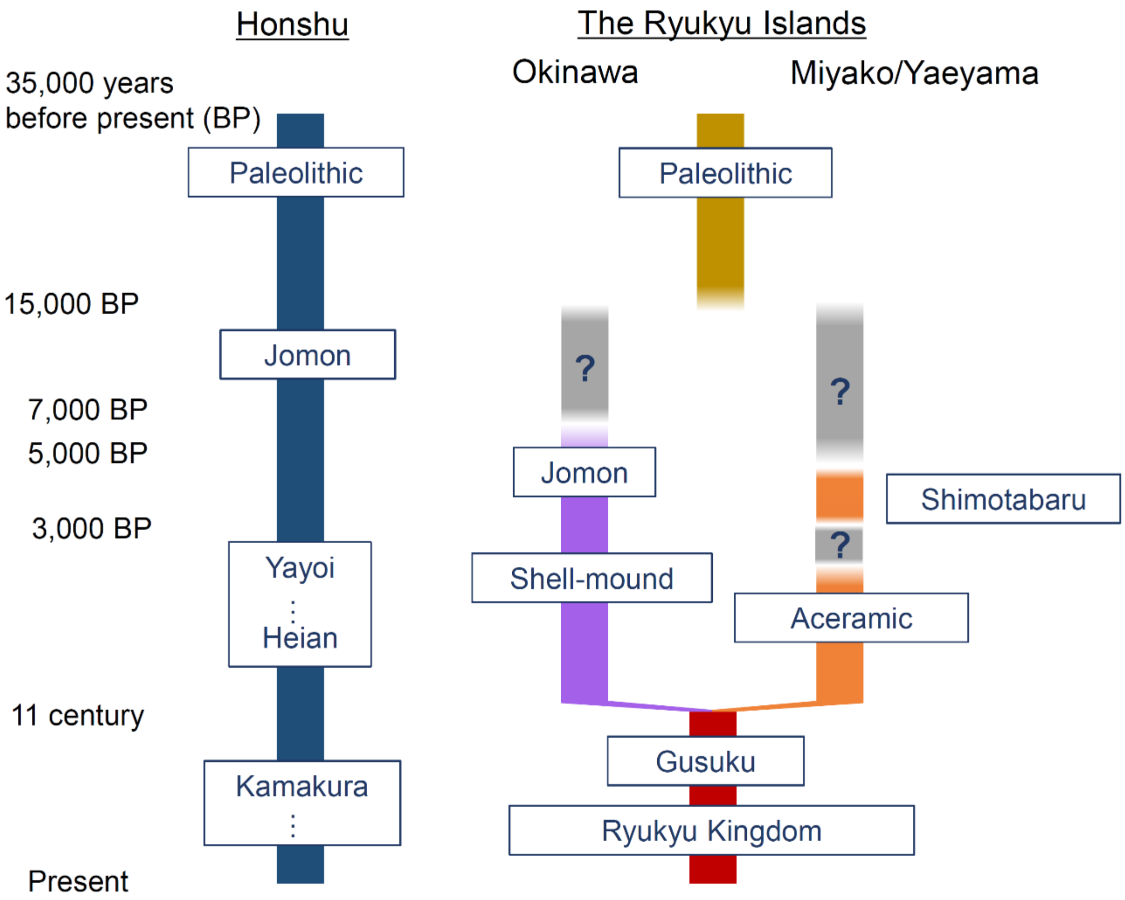


**Supplementary Figure 1** History of mainland Japan and the Ryukyu Islands.

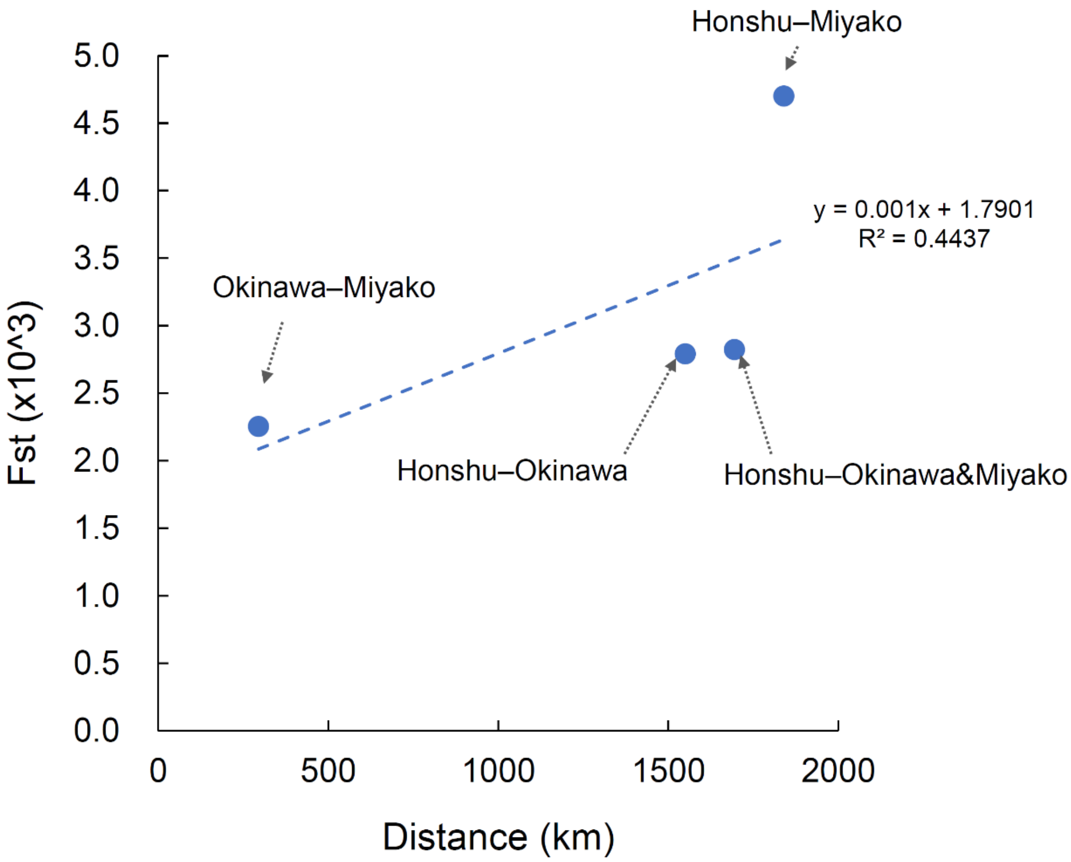


**Supplementary Figure 2** Correlation between $F_{ST}$ value and geographical distance.

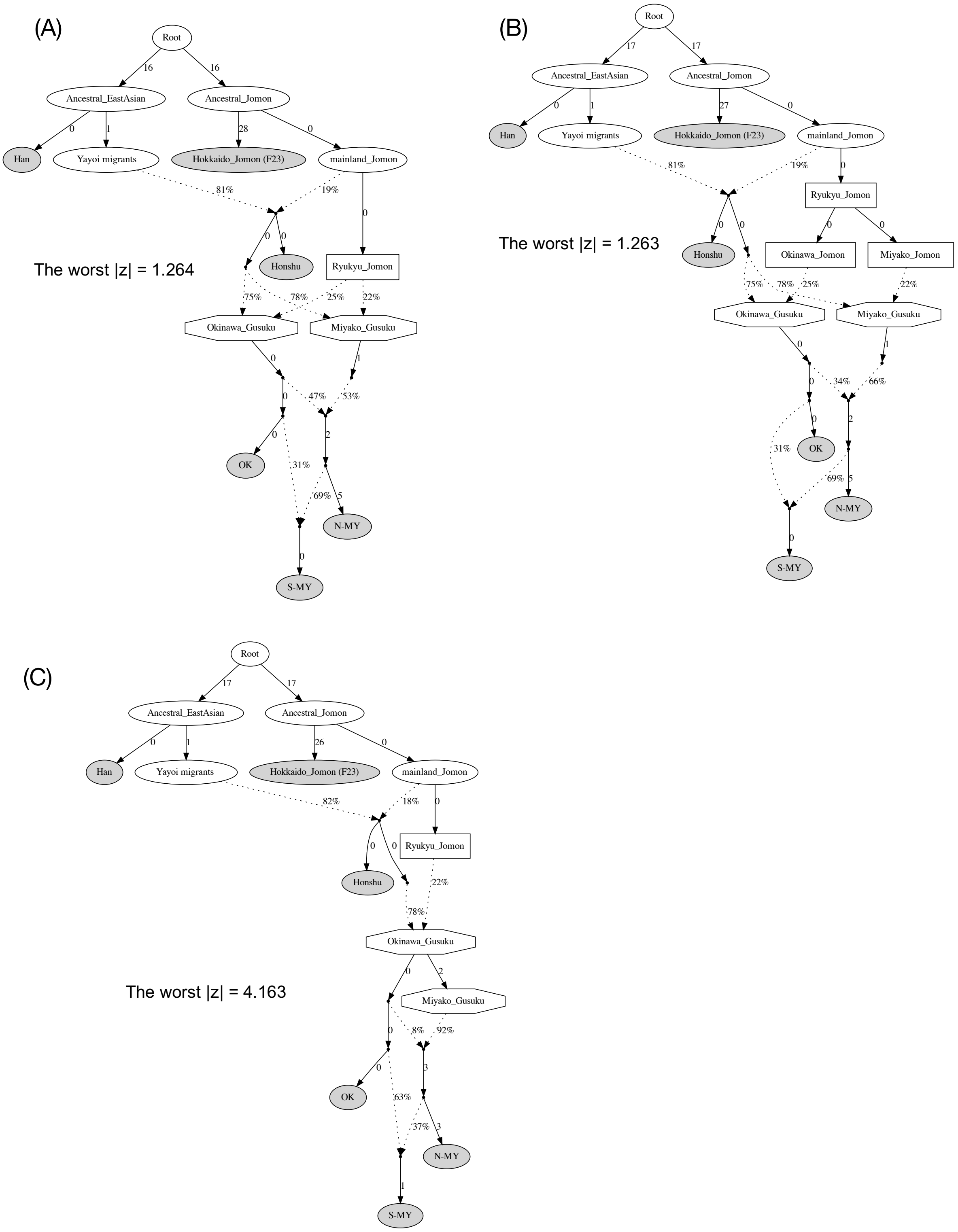


**Supplementary Figure 3** Admixture graph modeling for Ryukyu islanders using both of transversions and transitions. The three demographic models are the same as those in Fig. 4C–E.

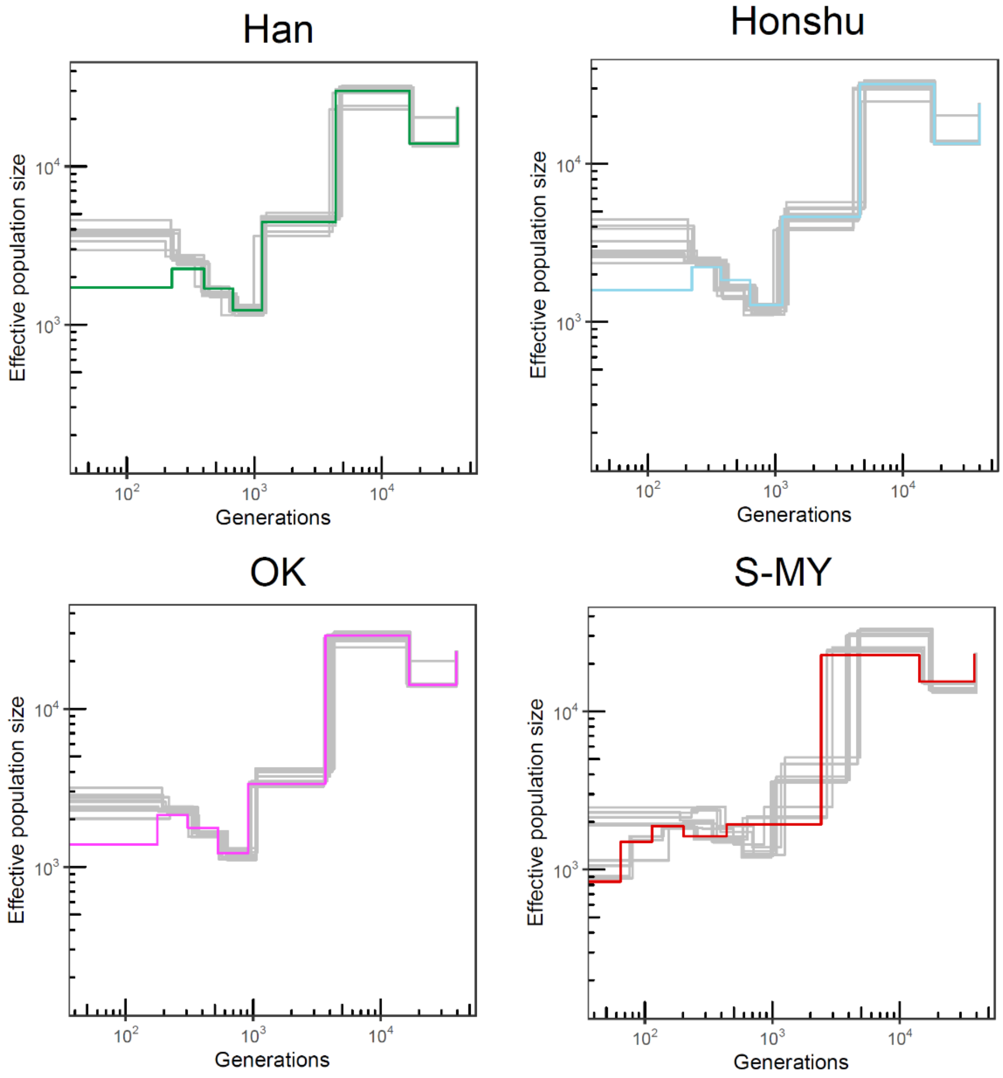


**Supplementary Figure 4** SMC++ estimation of effective population sizes using down-sampled datasets. Gray lines show the results of down-sampled (n=5) datasets. We confirmed that the bottleneck pattern in the N-MY cluster was not due to the smaller sample size.
